# Psychometric properties of questionnaires evaluating health-related quality of life and functional status in polytrauma patients with lower extremity injury

**DOI:** 10.1186/1752-2897-4-7

**Published:** 2010-06-28

**Authors:** Lian Jansen, Martijn PM Steultjens, Herman R Holtslag, Gert Kwakkel, Joost Dekker

**Affiliations:** 1 Department of Allied Health Care, St Antonius Hospital, Nieuwegein, The Netherlands; 2 Department of Rehabilitation Medicine and EMGO Institute, VU University Medical Centre, Amsterdam, The Netherlands; 3 Institute of Health and Wellbeing, Caledonian University, Glasgow, UK; 4 University Medical Centre Utrecht, The Netherlands

## Abstract

**Background:**

Long term disability is common among polytrauma patients. However, as yet little information exists on how to adequately measure functional status and health-related quality of life following polytrauma.

**Aims:**

To establish the unidimensionality, internal consistency and validity of two health-related quality of life measures and one functional status questionnaire among polytrauma patients.

**Methods:**

186 Patients with severe polytrauma including lower extremity injury completed the Sickness Impact Profile-136 (SIP-136), the Medical Outcomes Study 36-Item Short Health Survey (SF-36) and the Groningen Activity Restriction Scale (GARS) 15 months after injury. Unidimensionality and internal consistency was assessed by principal components analysis and Cronbach's alpha (α). To test the construct validity of the questionnaires, predetermined hypotheses were tested.

**Results:**

The unidimensionality and internal consistency of the GARS and the SF-36, but not the SIP-136 were supported. The construct validity of the SF-36, GARS and to a lesser extent the SIP-136 was confirmed.

**Conclusion:**

The SF-36 and the GARS appear to be preferable for use in polytrauma patients over the SIP-136.

## Introduction

People who sustain traumatic injury do not generally regain their pre-injury levels of physical functioning and experience difficulty in performing activities of daily living (ADL) [1,2]. Previous studies have suggested that the lower extremities are the most frequently injured body regions in polytrauma patients [3]. In addition, injuries of the lower extremities are believed to have a major impact on functional status and health-related quality of life (HRQoL) [3,4].

The Sickness Impact Profile-136 (SIP-136) [5] and the Medical Outcomes Study 36-Item Short Health Survey (SF-36) [6] are widely used measures of HRQoL and have been used in populations with a wide range of diagnoses and disease severity including trauma care [1,2]. The Groningen Activity Restriction Scale (GARS) [7,8] is a questionnaire, which measures patient's functional limitations of socially defined roles and tasks and is used in various countries in different populations. However, any instrument that is used to assess patients should have adequate psychometric properties and be appropriate for the patient population assessed. The reliability and validity of these questionnaires in trauma patients has not yet been established.

The aim of the present study was to investigate: 1) the unidimensionality, 2) internal consistency and 3) construct validity of the SIP-136, SF-36 and GARS among polytrauma patients with at least one injury of the lower extremity.

## Materials and methods

### Participants

The present study used data from a large prospective cohort study designed to examine multiple outcomes after traumatic injury [9]. Four hundred and ninety-nine consecutive severely injured patients who entered and stayed in the Hospital were considered for participation in this study. From this group, children below the age of 16 years (n = 40) were excluded, as were patients who died before the final assessment of outcome (n = 100). Of the 359 eligible patients, 335 gave informed consent and participated in the prospective cohort study. Twenty-four patients were lost to follow-up. Reasons for withdrawal from the study were: three lived abroad, seven addresses were untraceable, eleven patients withdrew their consent and three had an incomplete dataset.

For the present study patients were included if they had: 1) a fracture or injury of the lower extremity (including pelvis) with a Hospital Trauma Index (HTI) [10] of two or more, 2) an Injury Severity Score (ISS) of 16 or above and 3) age of 17 years or older and 4) were able to write and read Dutch. Individuals that had suffered spinal cord injury were excluded. This resulted in 186 patients being included in the analyses for the present study.

### Measures

Approximately 15 months after injury, patients completed the SIP-136, SF-36 and GARS.

The SF-36 [6] includes 36 multiple-choice items and takes approximately five to ten minutes to complete. The SF-36 is grouped into eight subscales scores: Physical and Social Functioning, Role Limitations due to Physical Health and Emotional Problems, Mental Health, Bodily Pain, Vitality and General Health. All raw scale scores are linearly converted to a 0 to 100 scale, with higher score indicating higher levels of functioning or well-being.

The SIP-136 [5] is a standardized questionnaire consisting of 136 (yes/no) statements about health-related dysfunction. The 136 items are grouped into 12 different categories: Ambulation, Mobility, Body Care and Movement, Social Interaction, Alertness, Emotional Behaviour, Behaviour, Communication, Sleep and Rest, Eating, Work, Home Management, Recreation and Pastimes. Each item is assigned a predetermined weight. Scores are calculated by summing the weights of all health related items and dividing by the maximum possible dysfunction score for each category. Scores are expressed as percentages, ranging from zero (no impairment) to 100 (maximum impairment).

Physical functioning was assessed by the GARS [7,8]. The GARS consists of 18 questions about daily activities, each with four response categories. Response choices range from 1) 'yes, I can do it fully and independently without any difficulty' to 4) 'no I cannot do it without someone's help'. The questionnaire comprises 11 items (scale range 11-44) referring to Activities in Daily Living (ADL, personal care) and seven items (scale range 7-28) to Instrumental Activities of Daily Living (IADL, household chores). The sum score provides information on the level of difficulty a person experiences in care taking and household activities. Sum scores may range from 18 (no disability) to 72 (maximum disability).

The following additional variables were assessed; age, gender, length of hospital stay, length of stay in Intensive Care Unit, discharge destination, pain and co-morbidity.

### Statistical analyses

To test the unidimensional structure of the subscales of the questionnaires, principal components analysis was applied. Items in the principal components analysis with loading less than 0.4 were considered inadequately representative of the underlying dimension. Analyses were performed separately per subscale. In the analysis of the SIP-136, three items were excluded because no patients scored positive on these items. To further explore the correlation between the subscales of the SIP-136 and SF-36 and GARS, the Spearman rank correlations between the subscale scores were calculated.

Internal consistency was investigated for all subscales of the SIP-136, SF-36 and GARS by calculating Cronbach's alpha (α).

Construct validity refers to the extent to which scores on a particular instrument relate to other measures in a manner that is consistent with theoretically derived hypotheses concerning the constructs that are being measured. To test the construct validity of the physical subscales of the SIP-136, SF-36 and GARS the following hypotheses were tested: HRQoL and functional status will be worse for 1) older patients, 2) patients with a longer hospital stay, 3) patients with a longer ICU stay, 4) patients who are discharged to other institutions instead of going home and 5) patients who experience more pain. Odds ratios (OR) and their 95% confidence intervals (CIs) were calculated using logistic regression. In all regression analyses, age and co-morbidity were included as control variables.

## Results

The characteristics of the study population are presented in table 1. Table 2 contains the mean, standard deviation, median, score range and proportion minimum and maximum score (floor and ceiling effect) of the (sub) scales of the questionnaires. All domains of the SIP-136 were skewed towards higher (worse) values. Large percentages of patients scoring the minimum (best) score indicating a floor effect. Fewer ceiling effects were measured with the SF-36 than for the SIP-136. Four scales of the SF-36 were skewed towards lower (worse) values with relatively large numbers scoring the maximum value (ceiling effect) on three of those four scales. The GARS Total, ADL as well as the IADL were skewed towards higher (worse) values with large percentages of patients scoring the minimum (best) score.

**Table 1:** Patient characteristics and clinical data measured 15 months after injury (n = 186)

**Gender **n (%)			
	Male	139	(74.7)
	Female	47	(25.3)
**Age **(years), mean ± SD (range)		38.4 ± 17.1	(17-87)
**ISS **mean ± SD (range)		24.5 ± 10.8	(16-66)
	Missing	1	
**Number of Surgery **mean ± SD (range)		2.6 ± 3.4	(0-32)
	Missing	3	
**Length of stay **(days), mean ± SD (range)			
	Intensive Care Unit	6.7 ± 14.2	(0-123)
	Hospital	27.9 ± 24.2	(1-153)
**Discharge destination **n (%)			
	Home	126	(67.7)
	Rehabilitation Institution or Care Home	60	(32.3)
**Co-morbidity **n (%)			
	None	91	(48.9)
	One or more	92	(49.5)
	Missing	3	
**Pain**^**1 **^mean ± SD (range)		3.0 ± 1.3	(1-6)

* n = number; SD = standard deviation; ISS = Injury Severity Score.

^1 ^Pain, range 1 - 6 (1 indicating no pain, 6 indicating extreme pain).

**Table 2:** Features of the score distributions of the SIP-136, SF-36 and GARS scales (n = 186)

Instrument Scale		Mean	SD	Median	Range^1^	Minimum^2^ (%)	**Maximum**^**3 **^**(%)**
**SIP-136**							
	Ambulation	17.32	17.8	15.3	0-67	40.3	0.5
	Mobility	6.76	13.6	0.0	0-60	67.7	0.5
	Body Care and Movement	5.92	10.1	1.5	0-55	46.8	0.5
	Social Interaction	7.73	13.2	0.0	0-73	52.2	0.5
	Alertness	12.69	20.9	0.0	0-97	59.7	0.5
	Emotional Behaviour	8.76	15.3	0.0	0-91	60.8	0.5
	Communication	4.08	9.8	0.0	0-53	80.1	0.5
	Sleep and Rest	9.46	13.8	0.0	0-78	54.3	0.5
	Eating	1.91	5.3	0.0	0-31	83.3	1.1
	Work	26.96	31.5	8.4	0-70	41.9	2.7
	Home Management	14.23	19.8	6.6	0-82	45.7	0.5
	Recreation and Pastimes	15.55	17.5	10.2	0-72	40.3	0.5
**SF-36**							
	Physical Functioning	62.90	29.6	65.0	0-100	2.7	12.4
	Role-Physical	53.20	42.7	50.0	0-100	30.1	36.6
	Bodily Pain	70.30	25.1	68.4	0-100	0.5	25.3
	General Health	67.10	20.6	70.0	10-100	1.1	2.7
	Vitality	62.40	19.1	65.0	0-100	0.5	1.6
	Social Functioning	74.80	28.2	87.5	0-100	4.3	34.9
	Role-Emotional	78.30	36.7	100	0-100	12.9	70.4
	Mental Health	74.30	19.6	80.0	12-100	0.5	4.3
**GARS**							
	Totaal	25.70	11.5	19.5	18-71	44.1	1.1
	ADL	14.50	6.0	11.0	11-43	51.6	1.6
	IADL	11.20	5.9	8.0	7-28	48.9	2.2

SIP-136 = Sickness Impact Profile-136; SF-36 = SF-36 item Health Status Survey; GARS = Groningen

Activity Restriction Scale; ADL = Activities in Daily Living; IADL = Instrumental Activities of Daily Living;

n = number; SD = standard deviation.

^1 ^Patients score range, minimum and maximum score.

^2 ^Percentage of patients with minimum score, floor effect.

^3 ^Percentage of patients with maximum score, ceiling effect.

Principal components analysis confirmed that the SF-36 and GARS were unidimensional. The domains of the SF-36 showed eigenvalues ranging from 1.6 to 6.7. The proportions of variance accounted for ranged from 51% to 89%. All items loaded adequately on their respective subscales (loading > 0.40). For the ADL scale of the GARS, all 11 items loaded on one component (eigenvalue of 6.82, percentage of variance accounted for was 62%). For the IADL score, also all items loaded on one component (eigenvalue of 5.3, percentage explained variance was 75.5%).

The unidimensionality of the SIP-136 was not supported. The 12 domains of the SIP-136 showed eigenvalues ranging from 1.9 to 6.0. The percentages of variance accounted for ranged from 24.6-43.2. Only three subscales had a factor loading > 0.40 of all items, whereas the other nine subscales had one or more (ranging from 1-12) items which were not loading on the scale (< 0.40).

Seven of the twelve subscales of the SIP-136 had a Cronbach's α higher than 0.70. The other five subscales had relatively small number of constituent items in a scale. All subscales from the SF-36 exceed the minimum required value of 0.70 for group comparison. The Cronbach's α of the total score of the GARS and the two subscales of the GARS range from 0.94-0.96.

To test construct validity forty logistic regressions models were computed, controlling for age and co-morbidity. Thirty-eight hypotheses were supported (p < 0.05) (table 3). The two not supported hypotheses concerned the SIP-136.

**Table 3:** Associations between patient characteristics and functional status and HRQoL 15 months after injury

		Age			Hospital stay		
		OR	95% CI	*P*	OR	95% CI	*P*
SIP-136	Body Care and Movement **	1.33	0.72 - 2.45	0.179	1.95	1.06 - 3.58	0.016
	Home Management *	2.30	1.60 - 5.62	0.001	4.58	2.32 - 9.04	0.000
	Mobility *	1.81	0.94 - 3.50	0.039	4.78	2.32 - 9.84	0.000
	Ambulation *	1.82	0.99 - 3.35	0.028	3.21	1.71 - 6.02	0.000
SF-36	Role-Physical **	0.44	0.24 - 0.82	0.005	0.34	0.18 - 0.64	0.001
	Physical Functioning *	0.35	0.18 - 0.66	0.001	0.16	0.08 - 0.33	0.000
GARS	ADL *	3.33	1.75 - 6.35	0.000	3.77	1.90 - 7.48	0.000
	IADL **	3.16	1.68 - 5.95	0.000	6.55	3.19 - 13.46	0.000
							
		**Intensive care stay**			**Discharge destination**		
		OR	95% CI	*P*	OR	95% CI	*P*

SIP-136	Body Care and Movement **	1.26	0.69 - 2.30	0.228	2.29	1,17 - 4,50	0,008
	Home Management *	2.79	1.43 - 5.42	0.002	3.36	1,64 - 6,87	0,001
	Mobility *	3.01	1.51 - 6.02	0.001	3.08	1,53 - 6,22	0,001
	Ambulation *	1.70	0.92 - 3.17	0.046	2.06	1,04 - 4,06	0,019
SF-36	Role-Physical **	0.51	0.27 - 0.95	0.018	0.36	0,18 - 0,72	0,002
	Physical Functioning *	0.35	0.18 - 0.68	0.001	0.31	0,15 - 0,62	0,001
GARS	ADL *	1.86	0.96 - 3.60	0.034	4.41	2,05 - 9,46	0,000
	IADL **	2.83	1.45 - 5.51	0.001	4.74	2,28 - 9,87	0,000
							
		**Pain**					
		OR	95% CI	*P*			

SIP-136	Body Care and Movement **	6.25	3.04 - 12.84	0.000			
	Home Management *	7.05	3.29 - 15.13	0.000			
	Mobility *	4.72	2.33 - 9.56	0.000			
	Ambulation *	8.73	4.00 - 19.08	0.000			
SF-36	Role-Physical **	0.10	0.04 - 0.23	0.000			
	Physical Functioning *	0.17	0.08 - 0.34	0.000			
GARS	ADL *	7.22	3.25 - 16.02	0.000			
	IADL **	5.72	2.74 - 11.95	0.000			

SIP-136 = Sickness Impact Profile- 136; SF-36 = SF-36 item Health Status Survey; GARS = the Groningen Activity Restriction Scale; ADL = Activities in Daily Living; IADL = Instrumental Activities of Daily Living; OR = Odds Ratio; CI = Confidence Interval.

*Adjusted for co-variates: age, gender and co-morbidity.

**Adjusted for co-variates: age and co-morbidity.

## Discussion

The results of our study support the reliability (unidimensionality, internal consistency) and construct validity of the SF-36 and GARS in a polytrauma population with lower extremity injury. Whereas the construct validity of the SIP-136 in this population was supported, the unidimensionality and internal consistency of the subscales are not supported in the present study.

The analysis of the GARS showed that the ADL and IADL scales can be used as separate (unidimensional) scales but the strong association between the two scales indicated that the scales do not measure different aspects of functional outcome. Other studies [7,11,12] also suggest one strong and reliable factor representing one underlying dimension of functional limitations.

Our study raises questions on the unidimensionality of most subscales of the SIP-136, suggesting that these subscales are not appropriate for use in a polytrauma population with injury of the lower extremity.

The internal consistency of the SIP-136 in the present study was low for most subscales. To our knowledge, little information about the internal consistency of the 12 separate scales of the SIP-136 is available in the literature. One study was found that reports sufficiently high Cronbach's α for the separate categories [13], while two other studies assessing the Cronbach's α of the subscales of the SIP-136 reported low Cronbach's [14,15]. High Cronbach's α from the total SIP-136 [5,13,16] and the physical and psychological dimension scores are reported [13]. However, Cronbach's α is dependent on the number of items in a questionnaire, a high α coefficient of the sum scores of the SIP-136 is therefore not surprising and not informative.

Our findings supported the construct validity of the SF-36 and GARS, these findings are comparable with the literature [3,11,17,18]. In our patient group, the construct validity of the SIP-136 was supported to a lower extent. Ho et al [16] found an advantage in using the SF-36 above the SIP because of its more robust construct validity, while others found some evidence to support the construct validity of the SIP [13,15,19].

The present study gives information about internal consistency and construct validity but does not provide information about other psychometric properties such as sensitivity to change over time and test-retest reliability. Additionally, other instruments may be suitable for this study population. Based on our results, further psychometric testing of the SF-36 and GARS in this population is recommended.

## Conclusion

The SF-36 and the GARS appear to be preferable for use in polytrauma patients over the SIP-136.

## Competing interests

The authors declare that they have no competing interests.

## Authors' contributions

LJ, MPMS and JD designed the study. LJ reviewed the literature and HRH collected all data.

LJ, MPMS and JD contributed to the analysis and interpretation of data which was initially performed by LJ. LJ drafted the manuscript, supported by MPMS. JD supervised and JD and GK reviewed the manuscript thoroughly for intellectual content.
